# OBJECTIVELY MEASURED SLEEP CHARACTERISTICS OF OLDER ADULTS WITH AND WITHOUT ALZHEIMER’S DISEASE

**DOI:** 10.1093/geroni/igz038.002

**Published:** 2019-11-08

**Authors:** Alex Laffer, Hilary J Hicks, Genna Losinski, Amber Watts

**Affiliations:** University of Kansas, Lawrence, Kansas, United States

## Abstract

Older adults commonly experience disturbed sleep such as difficulty initiating or maintaining sleep. Older adults who experience impaired sleep are at increased risk for cognitive decline or developing Alzheimer’s disease (AD). Research has shown that people with AD experience changes in sleep patterns, however, these changes are not well characterized. To better understand sleep in an older adult population with and without AD, the present study aimed to describe and compare objective sleep characteristics in both. Participants were older adults (126 with and 41 without AD) who wore an ActiGraph GT9X monitor on their non-dominant wrist for 7 days in a free-living environment. Results suggest that, compared to those without AD, participants with AD spent significantly more time in bed, t (165) = -4.37, p = .001), slept for longer durations, t (165) = -2.39, p = .044), and had less efficient sleep, t (165) = 2.71, p = .007. Participants with AD also had significantly greater sleep onset latency, more time awake after sleep onset, longer awakening lengths, and tended to arise later in the morning (all p ≤ .016). No differences were found between the groups in age, bedtime, or the number of awakenings during the night. These findings add to our understanding of the sleep disturbances experienced by older adults with and without AD. Significant group differences suggest that interventions may be necessary in treating sleep disturbances for older adults with and without AD. Future studies should examine sleep longitudinally to understand risk factors related to AD.

